# No Significant Role for Smooth Muscle Cell Mineralocorticoid Receptors in Atherosclerosis in the Apolipoprotein-E Knockout Mouse Model

**DOI:** 10.3389/fcvm.2018.00081

**Published:** 2018-07-09

**Authors:** M. Elizabeth Moss, Jennifer J. DuPont, Surabhi L. Iyer, Adam P. McGraw, Iris Z. Jaffe

**Affiliations:** ^1^Molecular Cardiology Research Institute, Tufts Medical Center, Boston, MA, United States; ^2^Department of Developmental, Molecular, and Chemical Biology, Sackler School of Graduate Biomedical Sciences, Tufts University School of Medicine, Boston, MA, United States

**Keywords:** smooth muscle cell, mineralocorticoid receptor, atherosclerosis, inflammation, apolipoprotein E, smooth muscle cell phenotype switching

## Abstract

**Objective:** Elevated levels of the hormone aldosterone are associated with increased risk of myocardial infarction and stroke in humans and increased progression and inflammation of atherosclerotic plaques in animal models. Aldosterone acts through the mineralocorticoid receptor (MR) which is expressed in vascular smooth muscle cells (SMCs) where it promotes SMC calcification and chemokine secretion *in vitro*. The objective of this study is to explore the role of the MR specifically in SMCs in the progression of atherosclerosis and the associated vascular inflammation *in vivo* in the apolipoprotein E knockout (ApoE^−/−^) mouse model.

**Methods and Results:** Male ApoE^−/−^ mice were bred with mice in which MR could be deleted specifically from SMCs by tamoxifen injection. The resulting atheroprone SMC-MR-KO mice were compared to their MR-Intact littermates after high fat diet (HFD) feeding for 8 or 16 weeks or normal diet for 12 months. Body weight, tail cuff blood pressure, heart and spleen weight, and serum levels of glucose, cholesterol, and aldosterone were measured for all mice at the end of the treatment period. Serial histologic sections of the aortic root were stained with Oil Red O to assess plaque size, lipid content, and necrotic core area; with PicroSirius Red for quantification of collagen content; by immunofluorescent staining with anti-Mac2/Galectin-3 and anti-smooth muscle α-actin antibodies to assess inflammation and SMC marker expression; and with Von Kossa stain to detect plaque calcification. In the 16-week HFD study, these analyses were also performed in sections from the brachiocephalic artery. Flow cytometry of cell suspensions derived from the aortic arch was also performed to quantify vascular inflammation after 8 and 16 weeks of HFD. Deletion of the MR specifically from SMCs did not significantly change plaque size, lipid content, necrotic core, collagen content, inflammatory staining, actin staining, or calcification, nor were there differences in the extent of vascular inflammation between MR-Intact and SMC-MR-KO mice in the three experiments.

**Conclusion:** SMC-MR does not directly contribute to the formation, progression, or inflammation of atherosclerotic plaques in the ApoE^−/−^ mouse model of atherosclerosis. This indicates that the MR in non-SMCs mediates the pro-atherogenic effects of MR activation.

## Introduction

The majority of myocardial infarctions and ischemic strokes are caused by rupture and thrombosis of atherosclerotic plaques, making atherosclerosis the leading cause of death worldwide (1, 2). Ample clinical data reveal that elevated levels of the hormone aldosterone are associated with an increased risk of myocardial infarction and stroke (3–5). Aldosterone is a steroid hormone that functions by activating the mineralocorticoid receptor (MR), a hormone-activated transcription factor. In the kidney, MR activation promotes sodium retention to regulate blood pressure. However, the increased risk of cardiovascular ischemia with elevated aldosterone appears to be independent of blood pressure (3, 5), supporting a potential pro-atherosclerotic role for extra-renal MR. Preclinical studies using animal models of atherosclerosis to explore this phenomenon have demonstrated that administration of aldosterone to apolipoprotein E knockout (ApoE^−/−^) mice accelerates atherosclerosis and plaque inflammation (6, 7). Conversely, pharmacologic MR blockade attenuates plaque development and inflammation in mouse (8–10), rabbit (11), and non-human primate models of atherosclerosis (12) through unknown mechanisms.

Atherogenesis begins with vascular damage induced by hypercholesterolemia and other cardiovascular risk factors. The damaged vessel activates pro-inflammatory pathways resulting in cytokine release and enhanced adhesion molecule expression on endothelial cells lining the vessel, thereby attracting inflammatory cells to the site of vascular damage. Macrophages in the nascent plaque phagocytose lipids to become foam cells in the plaque core. Additionally, smooth muscle cells (SMCs) migrate into and contribute to the developing atherosclerotic plaque in several ways. SMCs produce extracellular matrix components of the plaque fibrous cap, which stabilizes the plaque and prevents rupture (13). Later in plaque progression, some SMCs transform into osteogenic cells and contribute to plaque calcification (14), which correlates with risk of plaque rupture in humans (15). Finally, some SMCs within the plaque have recently been found to dedifferentiate and become inflammatory-like, losing expression of smooth muscle α-actin and instead expressing traditionally leukocyte-specific markers, even acting as foam cells themselves [reviewed in Bennett et al. 16]. Inflammatory cells in the plaque produce cytokines and reactive oxygen species to further promote plaque inflammation and produce matrix metalloproteases which can destabilize the fibrous cap. The resulting plaque rupture and thrombosis leads to ischemia of downstream tissues, which manifest as cardiovascular events such as myocardial infarction, ischemic stroke, and critical limb ischemia.

Clinical and animal data support that aldosterone and MR signaling promote plaque progression and increase inflammation, which may contribute to destabilization and rupture of the atherosclerotic plaque [reviewed in Moss et al. (17), Brown (18)]. However, the mechanisms underlying the contribution of the MR to plaque progression and inflammation have yet to be elucidated. In addition to the kidney, the MR is expressed in all cells of the vasculature, including in SMCs (19–21). When the MR is activated in SMCs in culture, these cells produce chemotactic cytokines that recruit inflammatory cells (7) and upregulate osteogenic factors that contribute to deposition of calcified material (22, 23). These *in vitro* studies support a potential role for SMC-MR in plaque inflammation and/or calcification. However, the contribution of SMC-specific MR to the development and phenotype of the atherosclerotic plaque has never been studied *in vivo*. In the present study, we developed an atheroprone mouse model in which the MR can be specifically deleted from SMCs in an inducible fashion in order to investigate the hypothesis that SMC-MR contributes to the development and progression of atherosclerosis and promotes vascular inflammation.

## Materials and methods

### Mouse models

All animals were handled in accordance with National Institutes of Health standards and all experiments were conducted with the approval of the Tufts Medical Center Institutional Animal Care and Use Committee. All mouse models were generated on the C57BL/6 background. Atheroprone mice with inducible SMC-specific MR deletion were generated by crossing our previously described MR^flox/flox^ × SMA-Cre-ERT2^+/−^ mice 20, 24, 25 onto the ApoE^−/−^ genetic background (Jackson Laboratories, Stock #002052). The Cre-ERT2 driver used in this mouse model is a ligand-dependent Cre recombinase construct whose activity is induced by selective estrogen receptor modulation via tamoxifen administration (26). The insertion of this Cre construct into the smooth muscle α-actin promoter produced mice containing a Cre recombinase that is transiently activated during tamoxifen administration only in smooth muscle α-actin-positive cells (SMA-Cre-ERT2^+/−^). ApoE^−/−^ × MR^flox/flox^ × SMA-Cre-ERT2^+/−^ mice and ApoE^−/−^ × MR^flox/flox^ × SMA-Cre-ERT2^−/−^ littermate controls were all treated with intraperitoneal injection of 1 mg of tamoxifen daily for 5 days at 6–8 weeks of age, prior to starting high fat feeding. The resultant male ApoE^−/−^ × MR^flox/flox^ × SMA-Cre-ERT2^+/−^ mice with SMC-specific MR recombination, hereafter referred to as “SMC-MR-KO”, and tamoxifen-treated ApoE^−/−^ × MR^flox/flox^ × SMA-Cre-ERT2^−/−^ littermates, hereafter referred to as “MR-Intact”, were used for all studies.

### PCR to confirm MR recombination

**Figure 1:**
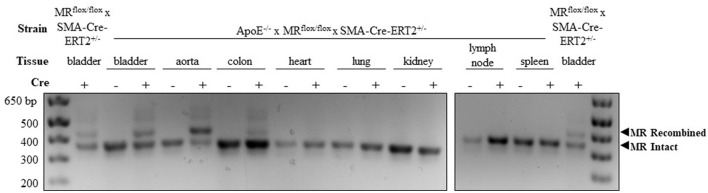
Confirmation of mineralocorticoid receptor (MR) gene deletion specifically in SMC-containing tissues in the atheroprone ApoE^−/−^ mouse model. Representative genomic DNA PCR products from tissues isolated 2 weeks after tamoxifen induction. MR recombination was tested in SMC-containing tissues (bladder, aorta, and colon) and SMC poor tissues (heart, lung, kidney, lymph node, and spleen) from SMA-Cre-ERT2^+/−^ animals and SMA-Cre-ERT2^−/−^ littermates. Bladder DNA from SMC-MR knockout mice without ApoE deletion (MR^flox/flox^ × SMA-Cre-ERT2^+/−^) served as a positive control. The recombined MR gene produces the larger 454 base-pair band and the LoxP-flanked intact MR gene produces the lower 364 base-pair band.

SMC-specific and nearly complete MR recombination in the vasculature has been previously confirmed in tamoxifen-induced MR^flox/flox^ × SMA-Cre-ERT2^+/−^ mice (24). To confirm SMC-MR recombination after crossing to the ApoE^−/−^ background, tissues were harvested from male SMA-Cre-ERT2^+/−^ and SMA-Cre-ERT2^−/−^ mice 2–4 weeks after tamoxifen induction. Genomic DNA was isolated using the Qiagen DNeasy kit. A PCR strategy was used such that LoxP-MR generates a smaller band (364 base pairs) while recombined MR yields a larger band (454 base pairs) as in Figure 1. The smooth muscle cell-containing bladder from MR^flox/flox^ × SMA-Cre-ERT2^+/−^ mice with intact ApoE was used as a positive control to compare the efficiency of MR recombination between the MR^flox/flox^ × SMA-Cre-ERT2^+/−^ and the ApoE^−/−^ × MR^flox/flox^ × SMA-Cre-ERT2^+/−^ double-KO. PCR was performed as previously described (24) with a combination of three primers:

5′-CCACTTGTATCGGCAATACAGTTTAGTGTC-3′,

5′-CACATTGCATGGGGACAACTGACTTC-3′,

5′-CTGTGATGCGCTCGGAAACGG-3′.

### Atherosclerosis protocols

Three experimental atherosclerosis protocols were used in this study. In the short-term early atherosclerosis protocol, tamoxifen-induced MR-Intact and SMC-MR-KO littermates were fed high fat diet (HFD, Envigo Teklad 88137, 42% calories from fat) for 8 weeks. In the long-term atherosclerosis protocol, mice were fed HFD for 16 weeks. In the aging atherosclerosis protocol, mice were allowed to age to 12 months on normal chow diet (Envigo Teklad 2918). All mice had free access to water. Mice were fasted for 4 h prior to tissue harvest and blood collection to measure fasting glucose (Nipro Diagnostics TrueBalance glucometer and strips) and cholesterol levels (Molecular Probes Amplex Red Cholesterol Assay Kit, Fisher). At the time of harvest, mice were weighed and then anesthetized with 2.5% isoflurane gas, blood was collected into heparinized syringes, and then the circulatory system was perfused with 0.9% sodium chloride solution via the left ventricle. The aortic arch was carefully isolated, cleaned, and separated from the heart approximately 1 mm from the aortic root and 2 mm distal to the left subclavian artery and stored on ice in PBS containing 0.5 mM EDTA until tissue digestion for flow cytometry (described below). In the 16-week HFD protocol, aortic arches were isolated for flow cytometry in one cohort, and in another cohort the brachiocephalic artery was removed at the level of the greater curvature of the aortic arch and frozen in OCT compound (Tissue-Tek #4583) for histology. The heart was first weighed then bisected horizontally at the level of the atria, and the upper portion of the heart was frozen in OCT for aortic root histology. The spleen was excised and weighed; the tibia was isolated and its length measured with calipers; the bladder was retained and snap-frozen for subsequent testing for MR gene recombination to confirm successful tamoxifen induction as needed.

### Measurement of tail cuff blood pressure and serum aldosterone levels

In the 5 days prior to animal sacrifice, tail cuff blood pressure was measured with the Kent Coda6 system using a protocol with 3 days of training and 2 days of measurement as we previously described and validated (27). To isolate serum, whole blood collected as above was incubated on ice for 1–4 h, centrifuged at 700 × *g* for 10 min, and the resulting serum transferred to a fresh tube. Aldosterone levels were measured using an Aldosterone Radioimmunoassay Kit (Tecan MG13051).

### Histology

Sequential cryosections of OCT-embedded aortic roots were cut such that all three leaflets of the aortic valve could be visualized. Cryosections of brachiocephalic arteries were cut sequentially from the origin of the artery from the aorta. Serial 10 μm sections were processed for staining with Oil Red O (ORO) as previously described (7) or for immunofluorescent staining with Alexa-594-conjugated anti-Mac2/Galectin-3 (Cedarlane, 1:500), FITC-conjugated anti-Acta2/smooth muscle α-actin (Sigma-Aldrich, 1:500), and DAPI (Fisher, 1:100) as previously described (28). Serial 6 μm sections were used for PicroSirius Red and Von Kossa staining. Immunofluorescent images were acquired using a Nikon A1R confocal microscope and brightfield images were acquired using an Olympus BX40 microscope and SPOT Insight camera and software. ORO-stained sections were analyzed to quantify plaque area, lipid content, and necrotic core area using ImagePro Premier v.9.0. All other stained sections were analyzed using ImageJ. Von Kossa staining was analyzed by a scoring method illustrated in Figure 9A, with 2–3 sections scored and averaged per mouse. All analyses were performed by genotype-blinded investigators.

### Flow cytometry

Immediately after animal sacrifice, aortic arches were minced with sharp scissors and digested with 125 U/mL collagenase type XI, 60 U/mL hyaluronidase type I-s, 450 U/mL collagenase type I (Sigma-Aldrich), and 60 U/mL *DNase-I* (New England Biolabs) in PBS with 20 mM HEPES in a 37°C shaker at 325 RPM for 1.5 h. The tissue was then ground through a 100 μm filter to obtain a single cell suspension and re-filtered through a 70 μm pipet-tip filter (Flowmi) to remove non-cellular debris. Cells were stained with APC-Cy7- or PE-conjugated anti-CD45.2, FITC-conjugated anti-CD3ε, APC-conjugated anti-CD11b, and PE-Cy7- or PE-conjugated anti-Ly6C antibodies (Biolegend, all 1:50), with Fc-block (BD Pharmingen, 1:50) added to the staining cocktail. After overnight storage at 4°C, stained cells were counted using a BD LSRII flow cytometer; data was captured with the BD FACS Diva software and analyzed using FlowJo v.10. Flow cytometry data from single-cell aortic arch suspensions were first gated on size to exclude cell debris and non-leukocyte populations. The resulting population was then gated on CD45+ status for quantification of total leukocytes. The CD45+ population was further separated into CD3+ and CD11b+ mutually exclusive populations. Within the CD11b+/CD3- population, the proportion of Ly6C negative, Ly6C-lo, and Ly6C-hi cells was determined. Flow cytometry measurements were performed in 2-3 independent experiments with all experimental groups represented in each study.

### Statistics

All mice that survived to termination of the study and developed atherosclerotic plaque were included in the analysis (one mouse was excluded due to no discernable plaque and one was euthanized due to poor health prior to study termination). Each study was performed in 2–3 batches of mice and the histologic analysis data were compared to the average value for the MR-Intact group for each batch. Data are reported as mean ± SEM. Means between two groups were compared with unpaired Student's *t*-test using GraphPad Prism v.7.01. Normally distributed data in Table 1 (blood pressure, weight, serum cholesterol, and serum aldosterone) were analyzed by 2-factor ANOVA with Holm-Sidak post-test using SigmaPlot v.12.5. Non-normally distributed data (fasting glucose, heart weight, and spleen weight in Table 1 and smooth muscle α-actin quantification in Figure 8B) were analyzed by Kruskal-Wallis ANOVA. The Von Kossa scoring data in Figure 9 was analyzed by Mann-Whitney Rank-Sum test using SigmaPlot v.12.5. Statistical significance was defined as *p* < 0.05.

**Table 1:** Cardiovascular risk factors and terminal measurements in MR-Intact and SMC-MR-KO mice on the ApoE^−/−^ background after 8 and 16 weeks of high fat diet.

**Duration of high fat diet**	**8 weeks**		**16 weeks**	
**Genotype**	**Intact**	**SMC-MR-KO**	**Intact**	**SMC-MR-KO**
Systolic BP (mmHg)	115.8 ± 3.2 (14)	113.0 ± 2.6 (15)	110.9 ± 3.1 (13)	109.9 ± 2.9 (10)
Diastolic BP (mmHg)	87.0 ± 3.0 (14)	83.7 ± 2.8 (15)	79.7 ± 2.9 (13)	80.9 ± 3.1 (10)
Weight post-HFD^$^ (g)	32.6 ± 1.3 (13)	32.1 ± 1.0 (14)	39.6 ± 1.4^#^ (18)	35.4 ± 1.4^*^ (14)
Fasting glucose (mg/dL)	94.1 ± 7.2 (14)	105.3 ± 11.4 (15)	145.5 ± 18.6 (15)	102.6 ± 8.9 (14)
Fasting cholesterol (mg/dL)	2153 ± 196 (8)	2124 ± 157 (10)	2097 ± 227 (10)	2219 ± 253 (10)
Serum aldosterone^$^ (pg/mL)	92.3 ± 16.0 (10)	112.4 ± 11.7 (12)	186.6 ± 23.2^#^ (10)	166.1 ± 17.1^#^ (10)
Heart weight^$^ (mg/mm tibia length)	7.54 ± 0.18 (11)	7.65 ± 0.22 (12)	8.74 ± 0.35^#^ (15)	8.16 ± 0.19 (14)
Spleen weight (mg)	110.0 ± 3.3 (11)	116.0 ± 4.2 (12)	113.2 ± 5.2 (16)	111.7 ± 4.3 (14)

BP, blood pressure; HFD, high-fat diet; SMC, smooth muscle cell; MR, mineralocorticoid receptor; SMC-MR-KO, smooth muscle cell-specific mineralocorticoid receptor knockout. Data are presented as mean ± SEM, with the N shown in parentheses for each measurement.

* p < 0.05 vs. treatment-matched MR-Intact;

# p < 0.05 vs. genotype-matched 8-week HFD;

$ *significant difference between 8- and 16-week HFD by ANOVA*.

## Results

### Confirmation of SMC-specific MR recombination in an atheroprone mouse model

In order to explore the role of SMC-MR in atherosclerosis, a well validated inducible SMC-specific MR knockout mouse (20, 24) was crossed to the atheroprone ApoE^−/−^ background. SMC-specific MR gene recombination on the ApoE^−/−^ background was confirmed using PCR of genomic DNA isolated from tissues collected from male tamoxifen-induced SMC-MR-KO and MR-Intact littermate controls. As shown in Figure 1, DNA from Cre-recombinase negative (MR-Intact) animals produced only the smaller PCR product, consistent with an intact floxed MR gene. The larger, 454 base pair recombined MR gene product is observed only with DNA from SMC-containing tissues from Cre-recombinase positive (SMC-MR-KO) animals that have been induced by tamoxifen. Specifically, bladder, aorta, and colon DNA produce both bands, consistent with MR recombination in these SMC-containing tissues. Compared to the colon, the aorta shows a greater proportion of the recombined MR band, consistent with a greater proportion of SMCs relative to endothelial and other non-SMCs, whereas the colon has a smaller proportion of SMCs and hence relatively more of the intact MR PCR product. Tissues in which SMCs are relatively scarce—heart, lung, kidney, lymph node, and spleen—did not produce evidence of MR recombination. Bladder DNA from the previously described SMC-MR-KO mouse without ApoE disruption (20, 24) served as a positive control for each PCR experiment. When compared to the SMC-MR-KO on the ApoE-intact background, the degree of recombination in the bladder was the same or greater on the ApoE^−/−^ background.

### SMC-MR deletion does not significantly alter aortic root plaque size or composition early in atherogenesis

**Figure 2:**
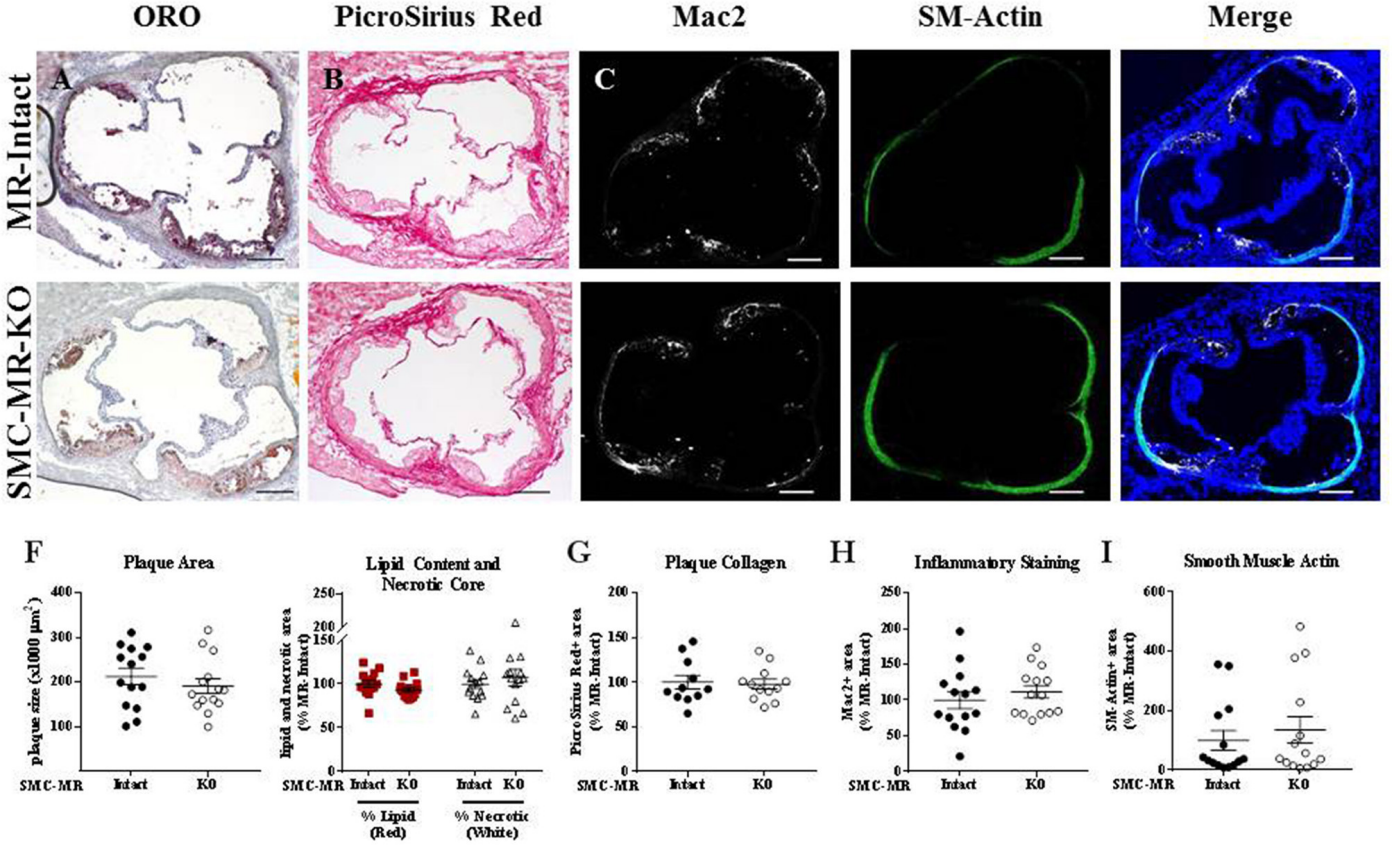
SMC-MR deletion does not alter aortic root plaque size or composition in young ApoE^−/−^ mice after 8 weeks of high fat diet. Representative histological images are shown for aortic root sections stained with: **(A)** Oil Red O (ORO); **(B)** PicroSirius Red for collagen; **(C)** Mac2/Galectin-3 immunofluorescence; and **(D)** Smooth muscle α-actin (SM-Actin) immunofluorescence, with the Mac2 and SM-Actin merged images shown in **(E)**. Plaque area is quantified in **(F)** along with plaque composition, including lipid content and necrotic core area. **(G)** Collagen content, **(H)** Mac2 positive area, and **(I)** SM-Actin positive area are quantified. Plaque composition data are expressed relative to the percent of each component in plaques from MR-Intact animals. All comparisons were not significant (*p* > 0.05) by unpaired student's *t*-test. Scale bars = 200 μm.

We first investigated whether SMC-MR influences early atherosclerotic plaque development by examining aortic root plaque size and composition after 8 weeks of HFD feeding in the ApoE^−/−^ model. Importantly, the absence of SMC-MR did not affect the degree of weight gain, blood pressure, serum glucose, cholesterol, or aldosterone levels at this time point (Table 1). Histology of the aortic root after 8 weeks of HFD feeding revealed no differences between SMC-MR-KO mice and MR-Intact littermates in atherosclerotic plaque size or in the percent of the plaque that is made up of lipids, necrotic core (Figures 2A,F,G), or collagen (Figures 2B,G). The degree of inflammatory staining within the plaque, as evidenced by Mac2 immunofluorescence (Figures 2C,H), was unchanged with SMC-MR deletion. We analyzed smooth muscle α-actin immunostaining within the plaque (Figures 2D,I) to identify those cells expressing this marker, and the levels of intra-plaque smooth muscle α-actin staining were also unaffected by SMC-MR deletion. It is important to note that this method does not identify all SMCs within the atherosclerotic lesion, nor does it identify only SMCs, as SMCs are known to downregulate SM-actin expression in atherosclerosis (28), and other cell types such as myofibroblasts may also express smooth muscle α-actin (29). Nevertheless, from these data we conclude that SMC-MR does not influence plaque accumulation or histologic indices of plaque vulnerability in the aortic root of ApoE^−/−^ mice after 8 weeks of HFD.

### SMC-MR does not contribute to vascular inflammation in ApoE^−/−^ mice after 8 weeks of HFD

**Figure 3:**
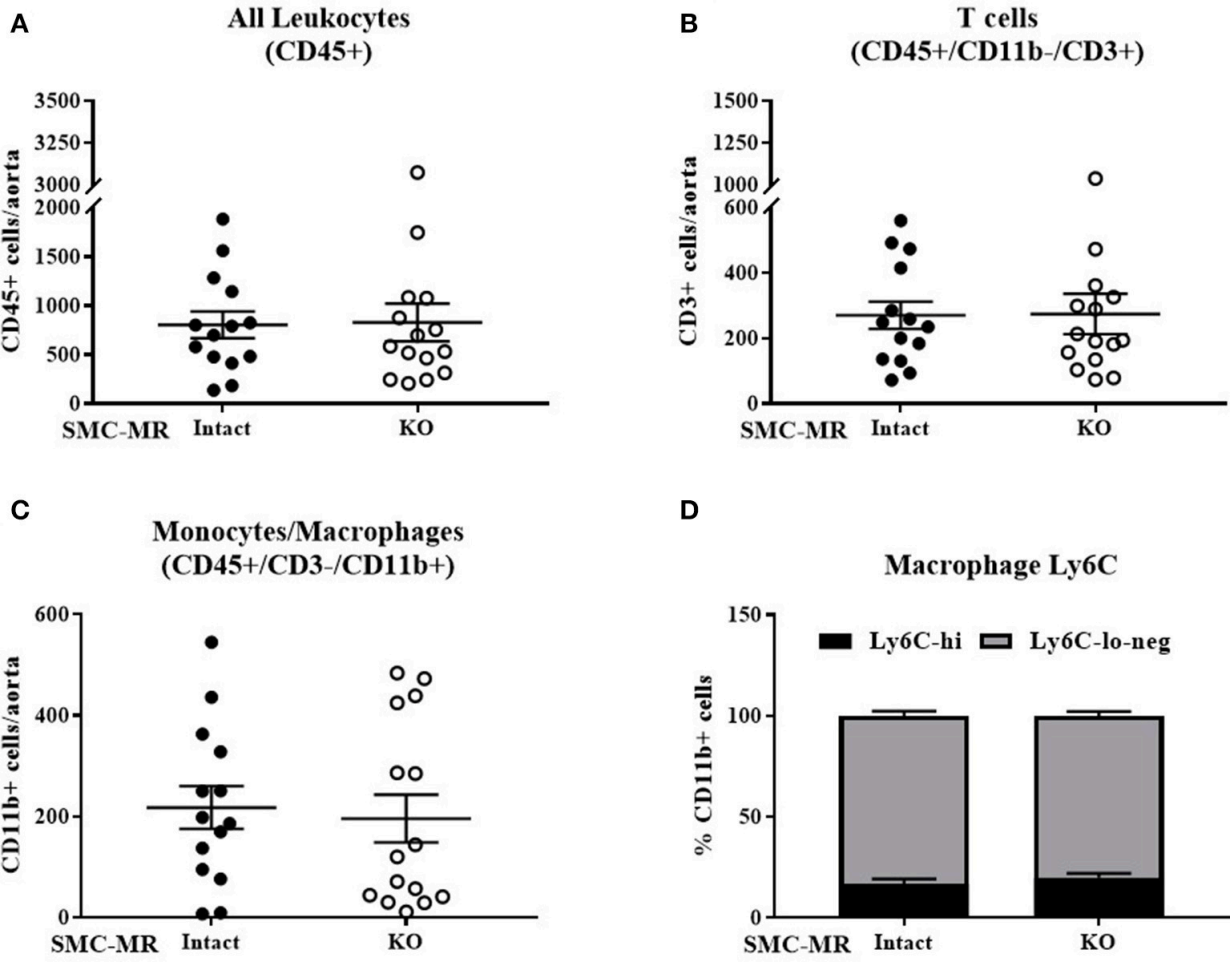
SMC-MR does not contribute to early vascular inflammation in ApoE^−/−^ mice after 8 weeks of high fat diet. The number of CD45+ leukocytes **(A)**, CD45+/CD11b-/CD3+ T cells **(B)**, CD45+/CD3−/CD11b+ monocytes and macrophages **(C)**, and the proportion of Ly6C-hi vs. Ly6C-lo-negative macrophages within the CD45+/CD3-/CD11b+ population **(D)** were quantified in the aortic arch by flow cytometry. All comparisons were not significant (*p* > 0.05) by unpaired student's *t*-test.

As plaque inflammation is highly correlated with plaque instability in humans (30), aortic arch inflammation was quantified using flow cytometry, a sensitive measure of vascular inflammation. After 8 weeks of HFD, freshly isolated aortic arches were digested to a single cell suspension and cells sorted to quantify the total number of CD45+ leukocytes, CD45+/CD11b-/CD3+ T cells, and CD45+/CD3-/CD11b+ cells within the vessel wall. Previous flow cytometry studies revealed that very few of the CD11b+ cells were neutrophils regardless of genotype (data not shown), thus we concluded that this population was predominantly monocytes and macrophages and further staining for neutrophils was omitted. As MR has been previously reported to contribute to macrophage phenotype (31), we further stratified CD11b+ cells into Ly6C negative, Ly6C-lo, and Ly6C-hi populations to differentiate between the more pro-inflammatory macrophage phenotype (Ly6C-hi) and the reparative macrophage phenotype (Ly6C-lo-negative) (32). SMC-MR deletion did not significantly influence the number of total leukocytes, T cells, or monocytes/macrophages in the aortic arch, nor did it alter the proportion of Ly6C-lo-negative vs. Ly6C-hi cells within the monocyte/macrophage population (Figure 3). From these data we conclude that SMC-MR does not play a significant role in vascular inflammation or atherogenesis after 8 weeks of high fat feeding, corresponding to early atherogenesis in the ApoE^−/−^ model.

### SMC-MR deletion does not significantly alter aortic root plaque size or composition in an aging model of atherosclerosis

**Figure 4:**
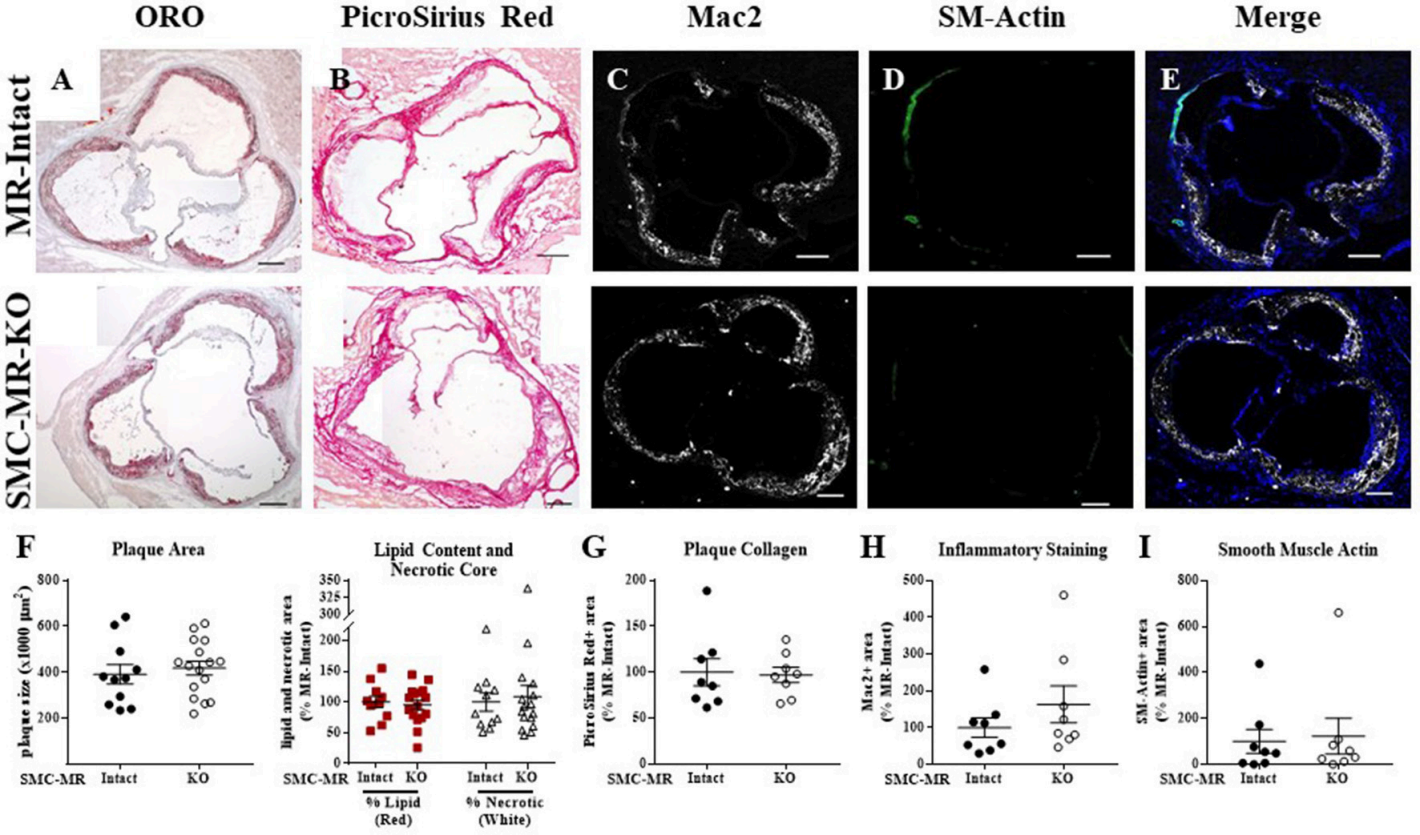
SMC-MR deletion does not alter aortic root plaque size or composition in ApoE^−/−^ mice aged for 12 months on normal chow diet. Representative histological images are shown for aortic root sections stained with: **(A)** Oil Red O (ORO); **(B)** PicroSirius Red for collagen; **(C)** Mac2/Galectin-3 immunofluorescence; and **(D)** Smooth muscle α-actin (SM-Actin) immunofluorescence, with the Mac2 and SM-Actin merged images shown in **(E)**. Plaque area is quantified **(F)** followed by plaque composition including: **(F)** Lipid content and necrotic core area **(G)** Collagen content, **(H)** Mac2 positive area, and **(I)** SM-Actin positive area. Plaque composition data are expressed relative to the percent of each component in plaques from MR-Intact animals. All comparisons were not significant (*p* > 0.05) by unpaired student's *t*-test. Scale bars = 200 μm.

In order to assess the potential role of SMC-MR in the slower atherogenesis that occurs when the ApoE^−/−^ mouse is allowed to age without high fat feeding, tamoxifen-induced SMC-MR-KO and MR-Intact littermates were aged to 12 months on normal chow diet. At 12 months of age, we observed a trend toward lower tail cuff blood pressure in the SMC-MR-KO mice that was not statistically significant (Table 2). There was also no difference in body, heart, or spleen weight, nor was there a difference in serum levels of glucose, cholesterol, or aldosterone between MR-Intact and SMC-MR-KO animals (Table 2). Histologic staining of aortic root sections likewise revealed no effect of SMC-MR deletion on plaque size, lipid content, necrotic core area, collagen content, Mac2 inflammatory staining, or smooth muscle α-actin staining in these animals (Figure 4). These data support that SMC-MR does not play a role in the development or progression of atherosclerotic plaques in the aging ApoE^−/−^ mouse model of atherosclerosis.

**Table 2:** SMC-specific MR deletion does not affect traditional cardiovascular risk factors in ApoE^−/−^ mice aged on normal chow for 12 months.

**12 months old normal chow**	**MR-intact**	**SMC-MR-KO**	***P*-value**
Systolic BP (mmHg)	121.4 ± 6.9 (7)	108.9 ± 4.7 (5)	0.1998
Diastolic BP (mmHg)	90.6 ± 4.9 (7)	83.0 ± 4.7 (5)	0.3078
Weight at 12 months (g)	35.5 ± 1.4 (12)	34.7 ± 0.8 (15)	0.6592
Fasting glucose (mg/dL)	213.1 ± 15.5 (11)	186.3 ± 25.5 (11)	0.3798
Fasting cholesterol (mg/dL)	1384 ± 267 (10)	1344 ± 171 (10)	0.9005
Serum aldosterone (pg/mL)	174.8 ± 32.2 (10)	223.2 ± 21.8 (10)	0.2299
Heart weight (mg/mm tibia length)	9.85 ± 0.29 (11)	9.89 ± 0.38 (15)	0.9419
Spleen weight (mg)	172.5 ± 69.4 (12)	103.4 ± 4.2 (15)	0.2752

*BP, blood pressure; SMC, smooth muscle cell; MR, mineralocorticoid receptor; SMC-MR-KO, smooth muscle cell-specific mineralocorticoid receptor knockout. Data are presented as mean ± SEM, with the N shown in parentheses for each measurement. All comparisons between MR-Intact and SMC-MR-KO mice were not significant (P > 0.05) by unpaired student's t-test*.

### SMC-MR–KO mice have in lower body weight after 16 weeks of HFD

Next we sought to determine whether SMC-MR influenced plaque characteristics in advanced disease, after 16 weeks of high fat feeding. As in previous experiments, we measured traditional cardiovascular risk factors as well as aldosterone levels and heart and spleen weights after this treatment regimen (Table 1). As expected, body weight in mice fed HFD for 16 weeks was significantly higher than that of mice fed HFD for 8 weeks. This increase in body weight was associated with higher aldosterone levels, as expected since the degree of obesity is known to correlate with increased levels of aldosterone in mice (33) and in humans (34). Other parameters were not significantly different between MR-Intact and SMC-MR-KO mice after 16 weeks of HFD. One exception was that there was a statistically significant (*p* < 0.05) 10% reduction in average body weight in SMC-MR-KO mice compared to MR-Intact controls after 16 weeks of HFD that was accompanied by a non-significant trend (*p* = 0.07) toward a reduction in fasting glucose levels in SMC-MR-KO mice compared to their MR-Intact littermates.

### SMC-MR deletion does not significantly alter aortic root plaque size, plaque composition, or vascular inflammation after 16 weeks of HFD feeding

**Figure 5:**
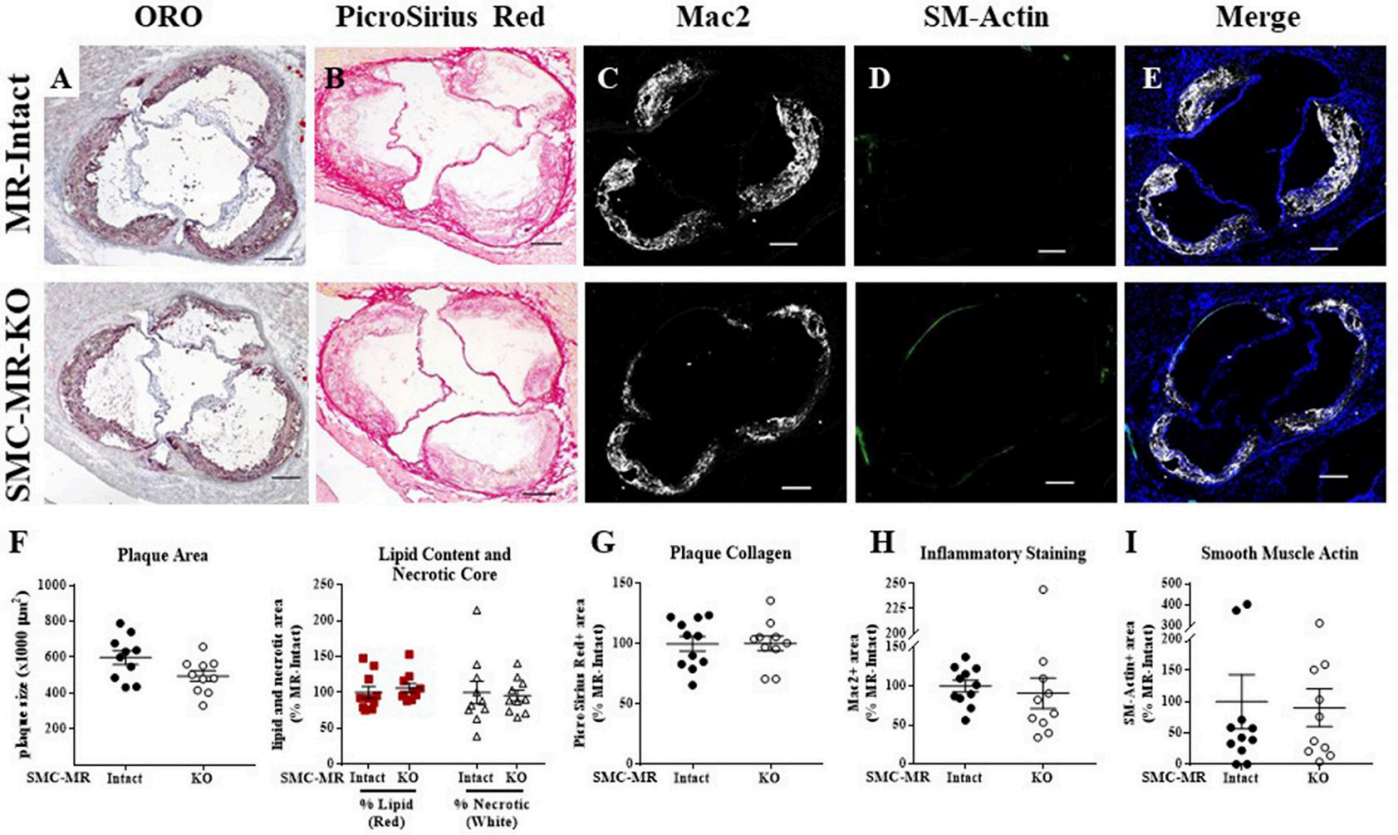
SMC-MR deletion does not significantly alter aortic root plaque size or composition in ApoE^−/−^ mice after 16 weeks of high fat diet. Representative histological images are shown for aortic root sections stained with: **(A)** Oil Red O (ORO); **(B)** PicroSirius Red for collagen; **(C)** Mac2/Galectin-3 immunofluorescence; and **(D)** Smooth muscle α-actin (SM-Actin) immunofluorescence, with the Mac2 and SM-Actin merged images shown in **(E)**. Plaque area is quantified **(F)** followed by plaque composition including: **(F)** Lipid content and necrotic core area **(G)** Collagen content, **(H)** Mac2 positive area, and **(I)** SM-Actin positive area. Plaque composition data are expressed relative to the percent of each component in plaques from MR-Intact animals. All comparisons were not significant (*p* > 0.05) by unpaired student's *t-*test. Scale bars = 200 μm.

**Figure 6:**
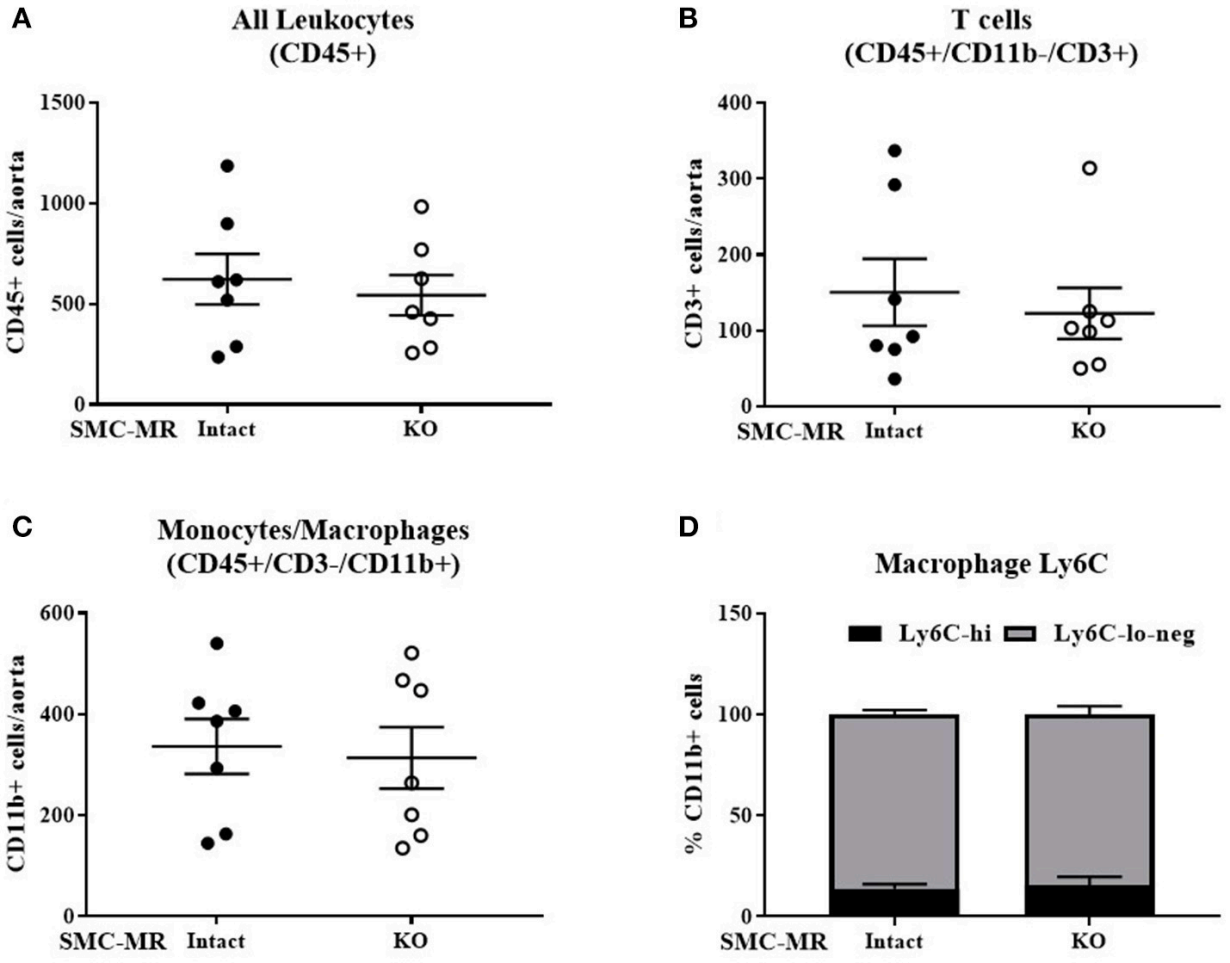
SMC-MR does not mediate advanced plaque inflammation in ApoE^−/−^ mice after 16 weeks of high fat diet. The number of CD45+ leukocytes **(A)**, CD45+/CD11b-/CD3+ T cells **(B)**, CD45+/CD3-/CD11b+ monocytes and macrophages **(C)**, and the proportion of Ly6C-hi vs. Ly6C-lo-negative macrophages within the CD45+/CD3-/CD11b+ population **(D)** were quantified by flow cytometry. All comparisons were not significant (*p* > 0.05) by unpaired student's *t*-test.

Histologic analysis of the aortic roots of MR-Intact and SMC-MR-KO ApoE^−/−^ mice fed HFD for 16 weeks revealed a trend toward a reduction in plaque area (Figures 5A,F left, *p* = 0.0503) in mice lacking SMC-MR, though this did not reach statistical significance. In addition, we observed no difference in plaque lipid content, necrotic core area (Figure 5A,F right), collagen content (Figures 5B,G), Mac2 inflammatory staining (Figures 5C,H), or smooth muscle α-actin staining between SMC-MR-KO and MR-Intact mice (Figures 5D,I). As expected, the smooth muscle α-actin staining was low in these advanced lesions, but this was unaffected by the absence of SMC-MR. We therefore conclude that SMC-MR does not significantly modulate aortic root plaque histologic parameters in a 16 week HFD model of advanced atherosclerosis. We next quantified by flow cytometry the leukocyte populations present in the aortic arches of MR-Intact and SMC-MR-KO ApoE^−/−^ mice following 16 weeks of HFD feeding. As in the 8 week study, there was no significant difference in the number of total leukocytes, T cells, or monocytes and macrophages in the aortae of SMC-MR-KO compared to MR-Intact mice, nor did we detect a difference in the proportion of Ly6C-hi versus Ly6C–lo-negative macrophages (Figure 6). We therefore conclude that SMC-MR does not affect the inflammatory profile of plaques in this late atherosclerosis model using a sensitive, quantitative flow cytometry analysis.

### SMC-MR deletion does not significantly alter brachiocephalic artery plaque size or composition after 16 weeks of HFD feeding

**Figure 7:**
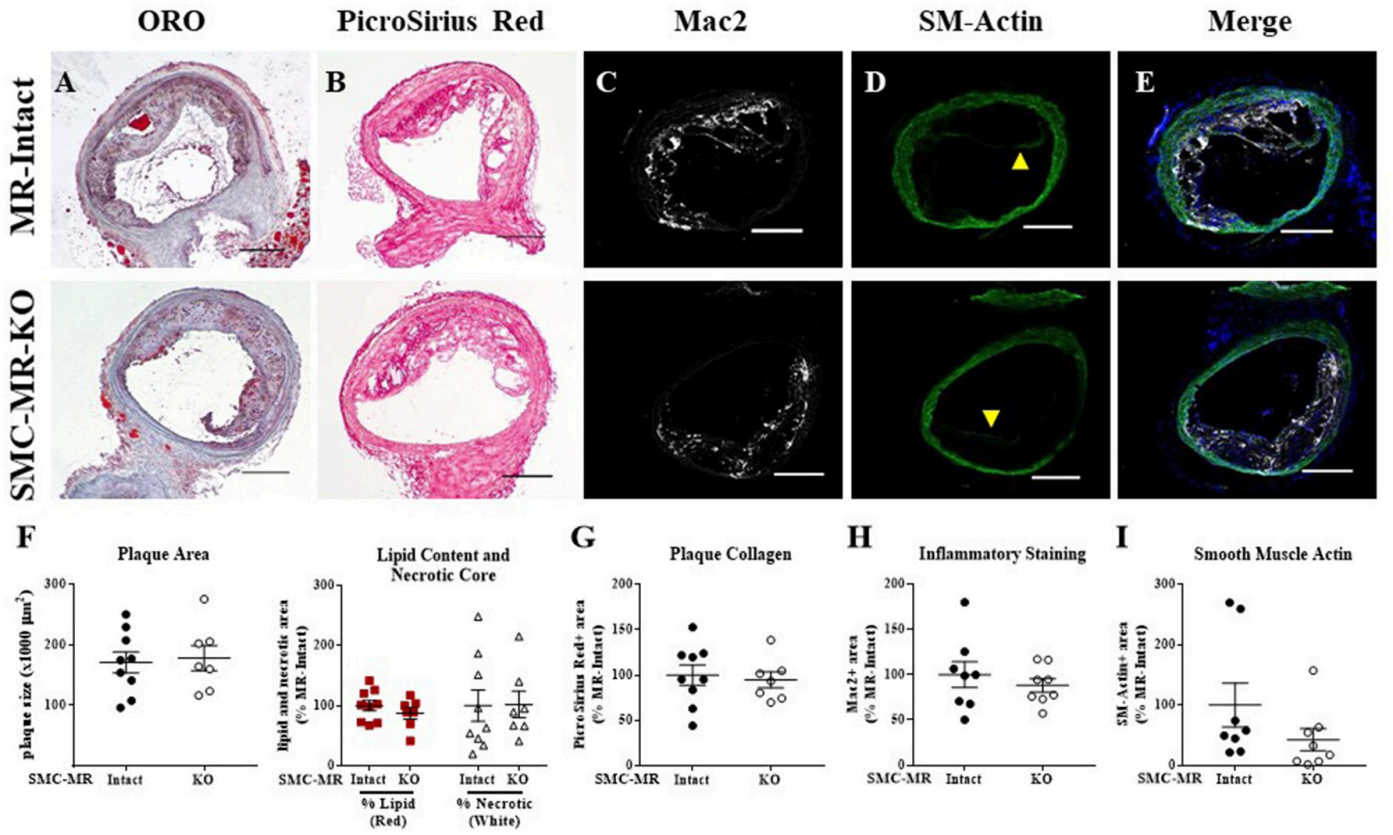
SMC-MR deletion does not significantly alter brachiocephalic artery plaque size or composition in ApoE^−/−^ mice after 16 weeks of high fat diet. Representative histological images are shown for brachiocephalic artery sections stained with: **(A)** Oil Red O (ORO); **(B)** PicroSirius Red for collagen; **(C)** Mac2/Galectin-3 immunofluorescence; and **(D)** Smooth muscle α-actin (SM-Actin) immunofluorescence (luminal fibrous cap staining marked by yellow arrowheads), with the Mac2 and SM-Actin merged images shown in **(E)**. Plaque area is quantified **(F)** followed by plaque composition including: **(F)** Lipid content and necrotic core area **(G)** Collagen content, **(H)** Mac2 positive area, and **(I)** SM-Actin positive area. Plaque composition data are expressed relative to the percent of each component in plaques from MR-Intact animals. All comparisons were not significant (*p* > 0.05) by unpaired student's *t*-test. Scale bars = 200 μm.

The data thus far demonstrate that in the ApoE^−/−^ model of atherosclerosis, deletion of MR specifically from SMCs does not significantly alter the size, composition, or degree of inflammation of atherosclerotic plaques in the aortic root under all 3 diet conditions (8 or 16 weeks of HFD or 12 months on normal chow). As SMCs can contribute to the formation of the fibrous cap, which stabilizes the plaque, we next investigated plaque characteristics in the brachiocephalic artery, a common anatomical location for analysis of advanced atherosclerotic lesions since plaques in this region can develop the classical lipid core with outward remodeling and fibrous cap formation that is typically not seen in the aortic root in the ApoE^−/−^ model (35). After 16 weeks of HFD, there was no difference in the brachiocephalic arterial plaques from SMC-MR-KO mice compared to their MR-Intact littermates in overall plaque size, lipid content, necrotic core (Figures 7A,F), collagen content (Figures 7B,G), Mac2 inflammatory staining (Figures 7C,H), or intra-plaque smooth muscle α-actin staining (Figures 7D,I). Of note, there is smooth muscle α-actin staining at the luminal surface of plaques in both groups, as shown in the representative images in Figure 7D (yellow arrowheads), indicating the presence of smooth muscle α-actin-positive fibrous cap formation in plaques of the brachiocephalic artery. However, when actin staining within the plaque was blindly quantified, there was no significant difference in staining in the brachiocephalic artery between SMC-MR-KO and MR-Intact mice (Figure 7I).

### Smooth muscle α-actin staining in the tunica media of the aortic root diminishes with advanced atherosclerosis, with no effect of SMC-MR deletion

**Figure 8:**
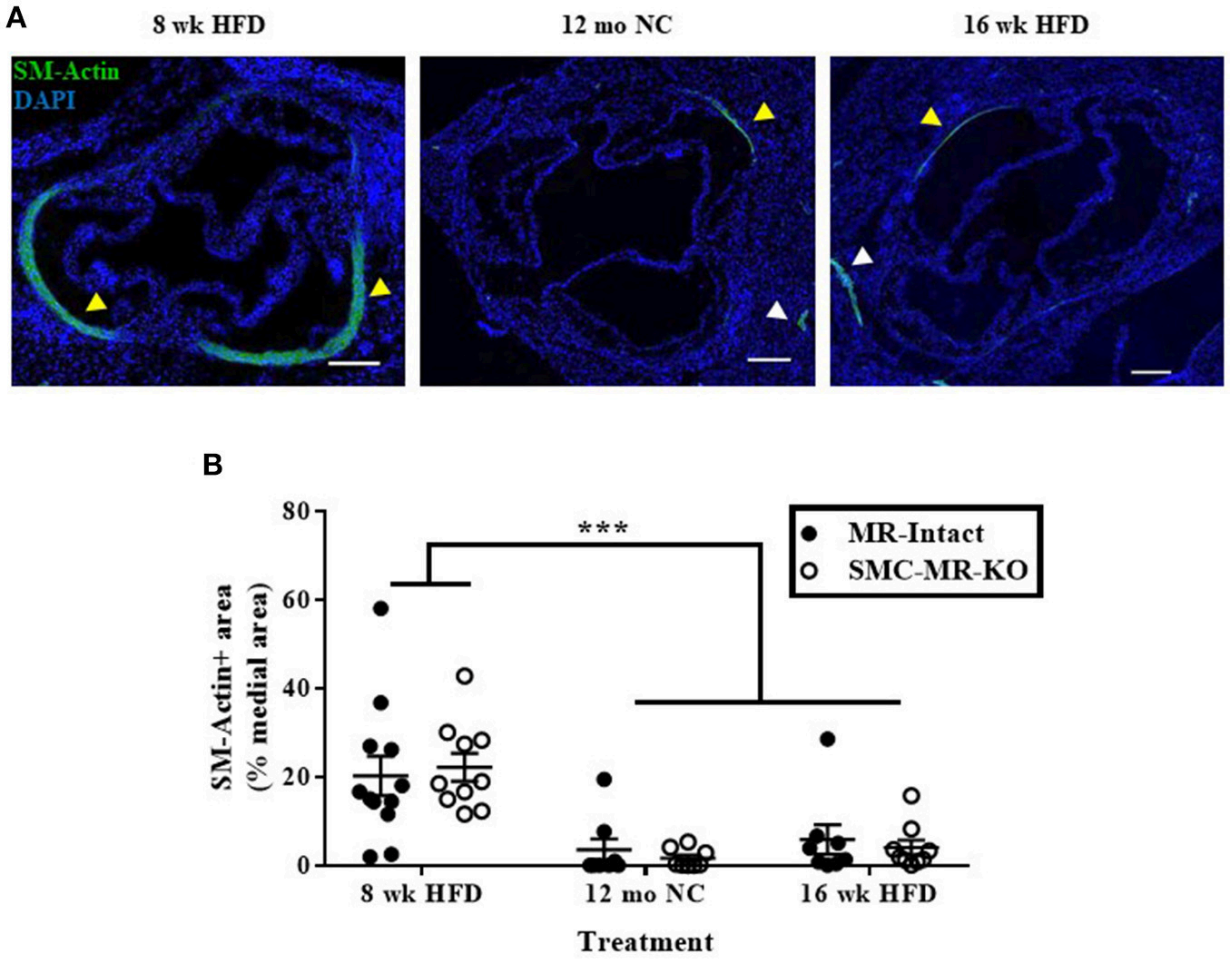
Smooth muscle α-actin staining in the tunica media of the aortic root diminishes with advanced atherosclerosis**. (A)** Representative immunofluorescent images displaying smooth muscle α-actin (SM-Actin) staining in the tunica media of the aortic root (yellow arrowheads) and in the walls of adjacent coronary vessels (white arrowheads) from mice fed high fat diet (HFD) for 8 weeks, normal chow (NC) for 12 months, or HFD for 16 weeks. Scale bars = 200 μm. **(B)** Quantification of SM-Actin positive staining relative to the total tunica media area of aortic root sections in SMC-MR-KO mice and MR-Intact littermates. ^***^p < 0.001 8 week HFD vs. 12 month NC and vs. 16 week HFD by Kruskal-Wallis ANOVA. There was no significant effect of genotype (*p* > 0.05). Scale bars = 200 μm.

As the plaque develops, SMCs de-differentiate from the contractile phenotype to the proliferative phenotype, losing expression of markers such as smooth muscle α-actin, and exhibit an alternative phenotype in which they migrate into the intima and proliferate. A subpopulation of smooth muscle α-actin-negative SMCs can even express inflammatory markers and may themselves contribute to atherosclerotic plaque instability (28). To consider more carefully whether the degree of de-differentiation of medial SMCs was modulated by the presence of SMC-MR, we further analyzed the smooth muscle α-actin staining in the tunica media of aortic root histologic sections. In these sections, we observed the expected decrease in smooth muscle α-actin staining in the tunica media of aortic roots from mice with more advanced atherosclerosis (after 12 months on normal chow or 16 weeks of HFD) compared with mice displaying earlier atherosclerotic lesions (8 weeks of HFD) (Figure 8A, yellow arrowheads), despite positive staining in nearby coronary vessels in adjacent regions of tissue (**white arrowheads**). The percentage of the media staining positive for smooth muscle α-actin was quantified and found to be significantly decreased in both the 12 month normal chow and 16 week HFD groups compared to in the 8 week HFD groups (Figure 8B). However, medial smooth muscle α-actin staining was not affected by the presence of SMC-MR. From this data we conclude that SMC-MR does not play a role in the decrease in smooth muscle α-actin expression in the tunica media with advancing atherosclerosis.

### SMC-MR deletion does not impact plaque calcification in the aortic root or the brachiocephalic artery after 16 weeks of HFD in the ApoE^−/−^ model

**Figure 9:**
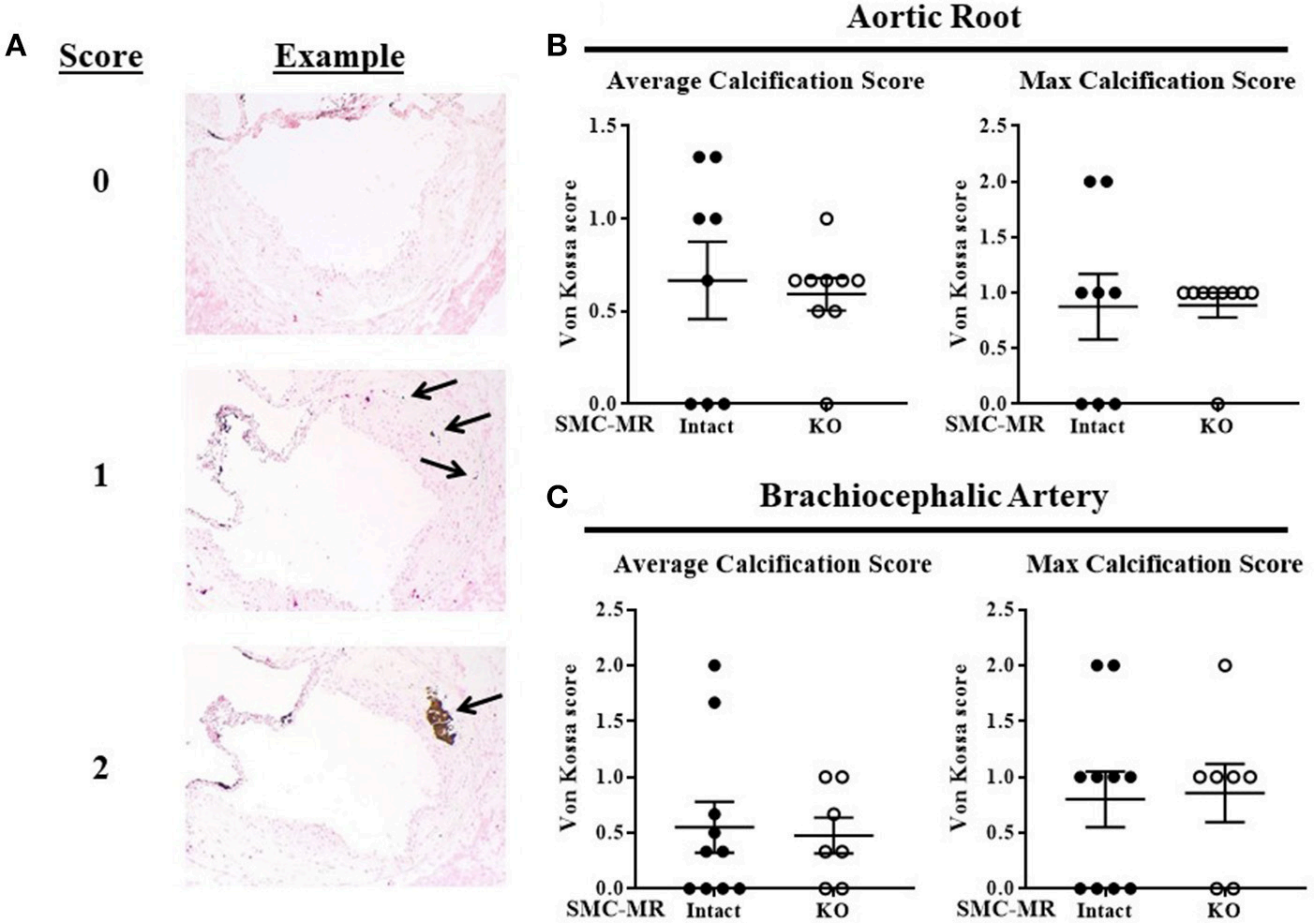
SMC-MR deletion does not impact plaque calcification in the aortic root or the brachiocephalic artery after 16 weeks of high fat diet. **(A)** Representative Von Kossa stained sections demonstrate the scoring method used. The average calcification score of three sections per mouse (left panels) and the maximum calcification score for each mouse (right panels) were compared between SMC-MR-KO and MR-Intact mice in the aortic root **(B)** and the brachiocephalic artery **(C)**. All comparisons were not significant (*p* > 0.05) by Mann-Whitney Rank Sum test.

As SMC-MR has been implicated in calcification in previous *in vitro* studies (22, 23), we examined whether SMC-MR plays a role in plaque calcification in these atherosclerosis models. As calcification is a late manifestation of atherosclerosis, there was no Von Kossa positive staining of aortic root sections in mice following only 8 weeks of HFD (data not shown), as expected in these early lesions. After 16 weeks of HFD feeding, Von Kossa staining of the aortic root and brachiocephalic artery sections was analyzed. Overall, very little Von Kossa positive staining was present even in these more advanced atherosclerotic lesions, therefore a scoring method was used to analyze the extent of calcification in these plaques. Representative images illustrating the scoring strategy can be found in Figure 9A. Using this scoring method, no significant difference in calcification was found between SMC-MR-KO compared to MR-Intact mice in either the average or maximum calcification score per mouse in either the aortic root (Figure 9B) or the brachiocephalic artery (Figure 9C).

## Discussion

This study explored, for the first time, the role of the MR specifically in SMCs in the process of atherosclerosis using the ApoE^−/−^ mouse model. This was addressed by generating a novel atheroprone mouse model in which the MR was deleted from SMCs in an inducible fashion on the ApoE^−/−^ background. The results reveal that the deletion of SMC-MR does not influence atherosclerotic plaque burden, composition (lipid, collagen, or necrotic core), or vascular inflammation after 8 or 16 weeks of high fat feeding or after 12 months of normal chow feeding in the ApoE^−/−^ model. We therefore conclude that the MR in SMCs does not contribute to atherosclerotic plaque development or progression in this model. Rather, the blood pressure-independent role of aldosterone in enhancing plaque development and inflammation, and conversely the capacity of MR antagonism to prevent atherosclerosis in mouse models, is mediated by the MR in cells distinct from vascular SMCs.

It is important to understand the role of the MR in atherosclerosis, as MR activation by aldosterone is strongly associated with the risk of cardiovascular ischemia, the leading cause of death. The rationale for exploring a specific role for SMC-MR in atherosclerosis was based on published reports showing that the MR contributes to the regulation of a variety of SMC functions that can contribute to atherosclerosis. Specifically, it has been shown *in vitro* that MR activation in vascular SMCs promotes vascular cell calcification (22, 23, 36) and release of chemokines and growth factors that promote leukocyte chemotaxis (7). Using tissue specific MR-KO mice, the MR in SMCs has also been shown *in vivo* to contribute to SMC proliferation after wire injury (37) and to vascular fibrosis and stiffening in response to injury, hypertension (38), and aging (25). The rationale for testing this in the ApoE^−/−^ mouse model is based on multiple studies showing that aldosterone enhances, and MR antagonists inhibit, atherosclerosis in this model (7, 8, 39). Despite these prior findings, we now demonstrate that in this atherosclerosis model, SMC-MR did not contribute to intimal calcification, SMC remodeling, or vascular inflammation. One reason for this paradox may be that SMCs *in vitro* display a profound phenotypic plasticity, taking on “phenotype-switched” characteristics such as proliferation, collagen synthesis, and α-actin downregulation simply from the nature of tissue culture (40), a feature which complicates *in vitro* study of SMC activities in atherosclerosis. Further, our findings suggest that the role of SMC-MR in the vasculature may be stimulus-specific. While we and others have found SMC-MR to play a role in the proliferative and fibrotic responses to carotid wire injury, hypertension, and aging, as mentioned above, the results of the present study would suggest that these vascular roles for SMC-MR do not extend to atherosclerosis, at least in the genetic ApoE^−/−^ model. Since MR generally has been shown to contribute to atherosclerosis and vascular inflammation specifically in this model (7, 8, 39), we conclude that these blood pressure-independent effects are mediated by non-SMC MR.

Ample data supports that MR activation by aldosterone is pro-inflammatory in atherosclerosis studies in animal models (7, 8, 11, 18). SMCs have been shown *in vitro* and in other disease models to exert pro-inflammatory effects via adhesion molecule upregulation, induction of oxidative stress, and production of pro-inflammatory cytokines and growth factors (41). We previously showed that the conditioned media from aldosterone-treated human coronary artery SMCs promotes chemotaxis of monocytes in a SMC-MR-dependent manner (7). Based on this finding, we hypothesized that SMC-MR would promote inflammation and plaque progression in the context of atherosclerosis. We tested this hypothesis using a novel SMC-MR-KO atherosclerosis model at various stages of plaque development, in multiple vascular beds, and using both conventional histology and quantitative flow cytometry analysis. However, despite our predictions drawn from the existing literature, SMC-MR deletion did not influence vascular inflammation in this model of baseline atherosclerosis in the absence of exogenous aldosterone administration. Notably, our study leaves open the possibility that SMC-MR could contribute to atherosclerosis exacerbated by aldosterone administration or other perturbations.

In humans, increased serum aldosterone is associated with increased risk of myocardial infarction and stroke (3–5), complications associated with the degree of atherosclerotic plaque inflammation (42). This is consistent with animal models in which aldosterone infusion increases plaque size and vascular inflammation (7, 39) and systemic MR inhibition reduces plaque burden and markers of inflammation (8, 10, 11). Synthesizing the current findings with this published data, we conclude that the MR acting in non-SMCs promotes plaque growth and inflammation. It was recently shown that deletion of the MR specifically from monocytes and macrophages reduces plaque size and histological markers of inflammation in both the angiotensin II-treated ApoE^−/−^ and the LDL receptor knockout mouse atherosclerosis models, supporting a role for the MR acting in inflammatory cells in atherosclerosis (43). Further, endothelial cells are known to be important for the initiation of atherosclerotic plaques and for the inflammation associated with the disease (17). *In vitro*, activation of endothelial cell MR has been shown to up-regulate expression of endothelial adhesion molecules, including intracellular adhesion molecule-1 (ICAM-1), thereby promoting leukocyte adhesion (44). *In vivo*, ICAM-1 has been shown to be necessary for aldosterone induction of atherosclerosis and vascular inflammation in the ApoE^−/−^ model (39). These data support the potential for endothelial cell MR to also contribute to the pro-inflammatory effects of MR activation in atherosclerosis. However, further studies are needed to test this *in vivo*.

Like inflammation, vascular calcification in humans is also associated with the risk of cardiovascular mortality. Calcification can occur in the intima, where it is associated with atherosclerosis, or in the media, where it is associated with vascular stiffness and valvular disease thereby contributing to hypertension and aortic stenosis (45). *In vitro* data strongly supports a role for the MR in regulating the osteogenic differentiation of SMCs, with MR activation promoting SMC calcification and MR inhibition preventing the process (22, 23, 36). Thus, while it appears from this study that SMC-MR may not contribute to intimal calcification *in vivo* in this atherosclerosis model, it remains possible that SMC-MR contributes to medial calcification commonly associated with renal failure and aging. Indeed, substantial recent data demonstrate that MR expression in vascular SMCs increases with age and contributes to hypertension and vascular stiffness in the aging vasculature (20, 24, 46). Further studies in models of medial vascular calcification are needed to test this possibility.

It has previously been described that SMCs de-differentiate during the development of atherosclerosis, losing expression of traditional SMC markers such as smooth muscle α-actin and in some cases, taking on a macrophage-like phenotype (16, 28). Direct treatment of SMCs in culture with oxidized phospholipids can recapitulate this effect (47). Further, renal denervation of ApoE^−/−^ mice, which resulted in a decrease in aldosterone levels, was shown to reduce atherosclerotic plaque burden and increase plaque smooth muscle α-actin staining (48). Further, *in vitro* studies have shown that SMC-MR contributes to SMC proliferation (49) while a recent *in vivo* study of rats with no cardiovascular risk factors indicated that MR activation promoted aortic collagen deposition, a hallmark of SMCs switching from the quiescent and contractile phenotype to the proliferative and synthetic (50). We have also demonstrated previously that SMC-MR is necessary for the SMC hyperplasia and collagen deposition observed after carotid wire injury (37). Consistent with prior reports, we observed very little smooth muscle α-actin positive staining within the aortic root plaques, and we observed a significant decrease in actin staining in the tunica media of mice with advanced disease, indicating that SMCs within the plaque and the media had lost their characteristic marker staining, indicating a switch in phenotype. However, the degree of smooth muscle α-actin staining was not affected by the presence of SMC-MR, indicating that the MR in these cells does not contribute to this phenomenon. It is important to note that this staining strategy does not identify all SMCs, nor does it differentiate between actin-positive SMCs and other actin-positive cell types, such as pericytes and myofibroblasts. Thus, although we see no role for SMC-MR, without more rigorous lineage tracing of SMCs, such as that described by Shankman et al. (28), we cannot definitively rule out the possibility that SMC-MR plays a role in pathogenic SMC phenotype switching in atherosclerosis.

Importantly, deletion of SMC-MR did not significantly affect blood pressure, fasting glucose or cholesterol levels under any of the conditions tested our experiments, which could have altered the results of the atherosclerosis studies independent of direct effects of SMC-MR. It is interesting to note that after 12 months of aging on normal chow, blood pressure tended to be lower in SMC-MR-KO mice compared to MR-Intact littermates, as we previously showed by telemetry in aged mice with intact ApoE (24). However, this was not statistically significant, likely due to the less sensitive tail cuff method of blood pressure measurement used in these studies. Surprisingly, we did observe a reduction in body weight and a trend toward a decrease in fasting glucose (*p* = 0.07) in SMC-MR-KO mice fed HFD for 16 weeks compared to MR-Intact littermate controls. These findings were not noted after 8 weeks of HFD or in 12 month old mice on normal chow. The trend toward a decrease in aortic root plaque area with SMC-MR deletion in the 16 week HFD study (*p* = 0.05) may be attributable to this significant difference in body weight, as obesity itself may be a risk factor for atherosclerosis (51). Potential mechanisms for this reduction in body weight with SMC-specific MR deletion are unclear. While aldosterone and MR signaling have been implicated in the components of the metabolic syndrome associated with obesity (52, 53), and MR blockade prevents obesity-induced metabolic syndrome in some animal models (54, 55), this relationship has not held true in human studies (56–58). Further, to our knowledge, no data currently exists linking SMC-specific MR to the development of obesity and metabolic syndrome. Thus, this area warrants further confirmation and investigation to characterize the possible link, if any, between SMC-MR and obesity.

Several limitations in this study must be acknowledged. First, we used endpoint PCR of genomic DNA, rather than more direct methods such as qRT-PCR or immunohistochemistry, to confirm SMC-specific MR deletion in our mouse model. This was due to the lack of available antibodies that are highly specific for mouse MR for immunohistochemistry, as the best MR antibodies that exist were raised in mouse (59). Instead, we confirm that genomic MR recombination is at least as efficient in the ApoE^−/−^ cross as in our previously extensively characterized SMC-MR-KO with intact ApoE that we include here as a control (24). As mentioned, tail cuff plethysmography was used to measure blood pressure instead of more sensitive telemetry measurements because the telemetry catheter in the aortic arch can affect blood flow characteristics thereby altering atherosclerosis. Thus, we cannot rule out small changes in blood pressure that are below the sensitivity of detection by this method. In addition, only male mice were used in this study, thus it remains possible that SMC-MR may influence atherosclerosis in females differently from males. Indeed, emerging evidence supports a sex difference in the contribution of the MR in other cell types to cardiovascular disease development and progression (60, 61). Thus the possibility of sex differences in the role of SMC-MR in atherosclerosis deserves further study. Finally, although the ApoE^−/−^ model of atherosclerosis is extensively used due to its development of plaques with similar composition to those of humans, no animal model completely reproduces the human disease. Importantly, the most commonly used atheroprone mouse models—ApoE^−/−^ and LDL receptor knockout—do not exhibit rupture of atherosclerotic plaques, necessitating the use of inflammation and plaque composition as proxies for plaque stability. There are also substantial differences between the various animal atherosclerosis models in terms of lipoprotein levels, gene expression, inflammation, and even the extent of lesion development (62). It is thus possible that our finding that SMC-MR does not play a role in atherogenesis is specific to the ApoE^−/−^ model and may differ if other models were tested.

Despite the limitations of this study, it is bolstered by several strengths. This investigation exhaustively and specifically explored the role of SMC-MR in atherosclerosis *in vivo* for the first time. Multiple plaque parameters were assessed including size, lipid content, necrotic core, smooth muscle α-actin content, and calcification by histologic methods and vascular inflammation was quantified by both conventional immunofluorescence and sensitive flow cytometry methods. These varied analyses were performed under three different treatment conditions (short-term HFD, long-term HFD, and aging on normal chow) and, for the 16 week HFD study, at two different locations in the vasculature (aortic root and brachiocephalic artery). Based on this thorough analysis, we conclude that the MR acting specifically in SMCs does not play a substantial role in plaque initiation, progression, or inflammation in the ApoE^−/−^ mouse model of atherosclerosis, and thus the MR in non-SMCs mediates the pro-atherogenic effects of MR activation in this model.

## Ethics statement

This study was carried out in accordance with the recommendations of the Guide for the Care and Use of Laboratory Animals, published by the National Institutes of Health. The protocol was approved by the Tufts Medical Center Institutional Animal Care and Use Committee.

## Author contributions

MEM performed experiments, analyzed data, and wrote the manuscript. JD performed experiments, analyzed data, and edited the manuscript. AM developed the mouse models and performed experiments. SI analyzed data. IZJ was involved in planning and oversight for all experiments, performed data analysis, wrote and edited the manuscript, and handled funding and regulatory requirements for all studies.

### Conflict of interest statement

The authors declare that the research was conducted in the absence of any commercial or financial relationships that could be construed as a potential conflict of interest. The reviewer DF and handling Editor declared their shared affiliation.
